# Effect of *Saururus chinensis* leaves extract on type II collagen-induced arthritis mouse model

**DOI:** 10.1186/s12906-018-2418-z

**Published:** 2019-01-03

**Authors:** Jong-Hyun Nho, Hyeun-Joo Lee, Ho-Kyung Jung, Ji-Hun Jang, Ki-Ho Lee, A-Hyeon Kim, Tae-Kyoung Sung, Hyun-Woo Cho

**Affiliations:** Tradition Korean Medicine Research Team, National Development Institute of Korean Medicine, 288, Jangheung, Seoul, 59338 South Korea

**Keywords:** *Saururus chinensis* leaves, Arthritis, Inflammation

## Abstract

**Background:**

*Saururus chinensis* leaves have been used as traditional medicine in Korea for pain, intoxication, edema, and furuncle. According to previous reports, these leaves exert renoprotective, neuroprotective, and antioxidant effects by attenuating inflammatory responses. However, the beneficial effect of *Saururus chinensis* leaves on arthritis has not been elucidated. Thus, we evaluated the water extract of *Saururus chinensis* leaves (SHW) using type II collagen-induced arthritis (CIA) mice models.

**Methods:**

Quantitative analysis of major components from SHW was performed by HPLC. Arthritis was induced by injection of type II collagen. Each group was orally administered SHW (100 mg/kg and 500 mg/kg). Methotrexate (MTX) was used as a positive control. Serum levels of interleukin-6, TNF-alpha, and type II collagen IgG in the animal models were measured using ELISA. Histological features were observed by H&E staining.

**Results:**

Quantitative analysis of SHW showed the contents as 56.4 ± 0.52 mg/g of miquelianin, 7.75 ± 0.08 mg/g of quercetin 3-*O*-(2”-*O*-β -glucopyranosyl)-α-rhamnopyranoside, and 3.17 ± 0.02 mg/g of quercitrin. Treatment with 500 mg/kg SHW decreased the serum level of Interleukin-6 (IL-6), TNF-alpha, and collagen IgG in the CIA model. Moreover, SHW treatment diminished the swelling of hind limbs and monocyte infiltration in blood vessels in CIA animal models. The results indicate that SHW could decrease CIA-induced arthritis in vivo.

**Conclusions:**

The results indicate that SHW could be used to improving arthritis by reducing inflammatory factors (IL-6 and TNF-alpha). However, further experiments are required to determine how SHW influences signal transduction in animal models.

**Electronic supplementary material:**

The online version of this article (10.1186/s12906-018-2418-z) contains supplementary material, which is available to authorized users.

## Background

*Saururus chinensis* is a perennial herbaceous plant belonging to the family Saururaceae. It is commonly found in South Korea, Japan, and China. As a traditional medicine in South Korea and China, *Saururus chinensis* leaves have been used for the treatment of pneumonia, edema, urination, and jaundice [1]. According to previous reports, the extract of *Saururus chinensis* (leaves, stems and flowers) shows renoprotective and antioxidant effects in rats fed a high-fructose diet [2], and inflammation-mediated neurotoxicity was found to be attenuated by treatment with the ethanol extract of *Saururi herba* in lipopolysaccharide (LPS)-stimulated BV-2 microglial cells [3]. These leaves also possess anti-asthmatic, anti-atopic, and anti-angiogenic activities [3–6]. In Korean traditional medicine, *Saururi herba* is used for the treatment of inflammatory diseases including fever, edema, and jaundice [7]. Sauchinone isolated from *Saururus chinensis* was found to reduce SREBP-1c-mediated hepatic steatosis and oxidative stress, as well as iron-induced liver injury [8, 9]. However, the beneficial effect of *Saururus chinensis* leaves on type II collagen-induced arthritis has not been elucidated.

Rheumatoid arthritis (RA) is one of the most common autoimmune diseases, affecting about 2% of the world population [10]. It is characterized by destruction of the cartilage and tendons, and inflammation in synovial joints. The pathophysiological mechanisms and causes of RA remain unclear, it is known that various immune cells including T- and B-lymphocytes, osteoclasts, fibroblast-like synoviocytes, and chondrocyte are involved in auto-immunity and chronic inflammation during RA pathogenesis [11, 12]. The type II collagen-induced arthritis (CIA) model is an animal model of rheumatoid arthritis that has been commonly used to validate therapeutic drugs based on the clinical and immunological features of RA [13]. Therefore, most drugs used for the treatment of RA have anti-inflammatory and anti-oxidative stress effects [14].

Inflammation is one of the defense mechanisms against body injury caused by infection. Inflammation disease presents symptoms such as fever and pain, and supports regeneration of damaged tissues [15, 16]. However, long-lasting inflammation responses induce neurodegenerative disease or inflammatory disease [17]. Inflammatory factors including intereukin-6 (IL-6), nitric oxide (NO), and prostaglandin E_2_ (PGE_2_) are secreted upon inflammation responses in the body, and these factors are used as indicators of inflammatory responses [18].

According to existing literature, current treatment strategies for RA are focused on improvement of joint damage and inflammatory response including swelling and fever. Thus, therapeutic agents for RA include glucocorticoids, specific inhibitors of inflammatory cytokines, and non-steroidal anti-inflammatory drugs (NSAIDs) [19]. However, most of these therapeutic agents induce adverse effects that influence the cardiovascular and gastrointestinal system, kidneys, and liver. Thus, searching for natural products with safety and efficacy in treating RA has become indispensable [20, 21]. In this study, we performed quantitative analysis of major components from *Saururus chinensis* leaves and examined the effect of SHW on RA using the type II collagen-induced arthritis (CIA) animal model.

## Methods

### Sample preparation and extraction

Thirteen dried *Saururus chinensis* leaf samples were purchased from Kyungdong market (Seoul, Korea). These samples were sourced from various regions and were identified by Professor Jong Gil Jeong (Plant taxologist, Dongshin University, Korea). A voucher specimen (TKM-II-7-1~13) of this plant was deposited in the Herbarium at the National Development Institute of Korea Medicine. We performed fingerprint and reproducibility analysis (Additional file 1:Supplementary Figure S1) for major components and selected seven samples. The same amount of *Saururus chinensis* leaves (3.1 kg, 443 g × 7) were extracted with water (30 L, 3 times) under reflux conditions for 3 h and filtered. The extracts were lyophilized using a freeze dryer (LYOPH-PRIDE 20R, IlShinBioBase, Dongducheon, Korea) to obtain SHW powder (652 g, yield: 21%). The lyophilized powder was dissolved in 0.5% carboxymethyl cellulose (CMC, Sigma-Aldrich, USA) before being used in experiments.

### LC-IT-TOF-MS conditions

Electrospray ionization-mass spectra were obtained on a LC-IT-TOF mass spectrometer (Shimadzu, Japan). All solvents used for analyses were of HPLC grade and were purchased from J.T. Baker (PA, USA). LC analysis was performed on a Shimadzu analytical UFLC (Kyoto, Japan) system comprising two LC-30 AD XR pumps, a CTO-20A column oven, a DGU-20A3 degasser, an SPD-20A detector, and an SIL-20A XR auto-injector. For full scan MS analysis, spectra were recorded in the range *m/z* 100–1000. Data were later processed using LC/MS solution software (version 3, Shimadzu, Kyoto, Japan), which includes a formula predictor. Detailed analytical conditions are listed in Table 1. SHW (50 mg) was dissolved in 5 mL of 70% methanol and filtered through a 0.2 μm syringe filter (Adventec, Canada) for the analysis.

**Table 1:** LC-IT-TOF MS conditions for *S. chinensis* extract

HPLC condition			
Column	BEH C_18_ (1.7 μm, 2.1 × 150 mm)		
Flow rate	0.21 mL/min		
Injection volume	1 μL		
Column temperature	40 °C		
Mobile phase	A: 0.1% formic acid in water B: acetonitrile		
	Time	A (%)	B (%)
	0	100	0
	25	55	45
MS condition			
Ionization mode	ESI, positive		
Capillary voltage (kV)	4.5		
CDL voltage (V)	10		
Detector voltage (kV)	1.67		
CDL temperature	200 °C		
Heat block temperature	200 °C		
Nebulizing gas	N_2_, 1.5 L/min		
Collision gas	Ar		
Collision Energy	30%		

### Isolation and identification

Dried leaves of *Saururus chinensis* (300 g) were suspended in 3 L water containing 2% acetic acid and then partitioned with *n*- hexane, methylene chloride and *n*-butanol, yielding 2 g, 3.5 g, 65 g, 205 g, respectively. The BuOH fraction (1 g) was dissolved in 10 mL of methanol and purified using a Luna C_18_-100A column (Phenomenex, USA; 25 cm × 3 mm, 5 μm particle size) on an LC20AP series high-performance liquid chromatography (HPLC) system equipped with SPD-M20A (Shimadzu, Tokyo, Japan). The mobile phase consisted of 0.02% formic acid in water (A) and acetonitrile (B), starting with 10% B increasing to 30% B for 45 min. The flow rate was 35.0 mL/min and the wavelength was 254 nm. The fraction was purified as an eluent to yield pure compound **2** (18.5 min, 70 mg) and **3** (21.1 min, 200 mg). NMR spectra were obtained using a Varian UNITY INOVA 500 NMR spectrometer operating at 500 MHz (^1^H) and 125 MHz (^13^C) using CD_3_OD (Sigma-Aldrich, USA); chemical shifts are given in ppm (δ).

### Qualitative analysis

SHW powder (200.0 mg) was extracted respectively with 45 mL of 0, 30, 50, 70, and 100% methanol under sonication for 60 min at room temperature. The extract was filtered, adjusted to a final volume of 50 mL in a volumetric flask, and then filtered through 0.2 μm syringe filter. Quercitrin purchased was Sigma-Aldrich (St. Louis, MO, USA). Miquelianin and quercetin 3-*O*-(2”-*O*-β-glucopyranosyl)-α-rhamnopyranoside [Q-3-(2″-glu)-rham] were isolated from *Saururus chinensis* leaves. A mixture of miquelianin, Q-3-(2″-glu)-rham and quercitrin was prepared in 70% methanol and serially diluted (concentration: 420–26.25 μg/mL, 105–6.25 μg/mL, 58.75–3.67 μg/mL) to obtain calibration curves. The chromatographic system used was an Agilent 1200 series HPLC system (Agilent, USA) with an MG II column (C_18_, 4.6 × 250 mm, Shiseido, Japan). The mobile phase was a gradient of 0.1% formic acid in water (A) and 0.1% formic acid in acetonitrile (B). The linear gradient elution was 15% of B to 25% of B for 0–30 min at a flow rate of 1.0 mL/min. The column oven temperature was 40 °C, and the wavelength was 254 nm.

### Animals and euthanasia

Male DBA/1 mice (6 weeks of age) purchased from Samtako (Osan, Korea) were separated into 5 groups (Control; *n* = 7, CIA; n = 7, CIA + MTX; n = 7, CIA + 100 mg/kg SHW; n = 7, CIA + 500 mg/kg SHW; n = 7). Immunization grade bovine type II collagen (Chondrex, Redmond, WA, USA) was dissolved in complete Freund’s adjuvant (CFA, Sigma Aldrich, St. Louis, MO, USA) or incomplete Freund’s adjuvant (IFA, Sigma Aldrich, St. Louis, MO, USA). The first immunization was performed using bovine type II collagen dissolved in CFA (1:1 ratio), mice were injected at the base of the tail (100 μl). After one week, bovine type II collagen dissolved in IFA (1:1 ratio) was injected at the base of the tail (100 μl) [22]. After 1 week of the second immunization, the CIA + MTX group was administered with methotrexate (0.2 mg/kg, p.o., once a day, 3 weeks). The CIA + 100 mg/kg SHW group was administered with 100 mg/kg SHW (p.o., once a day, 3 weeks). The CIA + 500 mg/kg SHW group was administered with 500 mg/kg SHW (p.o., once a day, 3 weeks). At the end of the experiment, mice were euthanized by carbon dioxide (CO_2_) inhalation according to the standard laboratory operating procedures of the IACUC.

### Assessment of arthritis and histologic score

Clinical arthritis was assessed weekly beginning from 21 days of the second immunization, and arthritic scoring was performed by three independent examiners, three times per week. Arthritis scoring was performed as described by Endale et al. [23]. The clinical assessment was as follows: 0 = symptomless, 2 = erythema, 4 = mild swelling and erythema, 6 = mild swelling, erythema from the tarsals to the ankle, 8 = moderate swelling, erythema from the metatarsal joints to the ankle, 10 = severe swelling and erythema from the foot to the ankle. Histological sections were stained with hematoxylin and eosin, and analyzed microscopically by three observers for the degree of inflammation and bone erosion according to the method reported previously [24, 25]. The following scale was used: 0 = normal, 1 = cell infiltration in synovial membrane, 2 = cartilage erosion, 3 = erosion of subchondral bone, and 4 = loss of joint integrity and ankylosis.

### Measurement of type II collagen IgG

Measurement of type II collagen IgG in serum was performed using Mouse anti-mouse type II collagen IgG antibody assay (2036, Chondrex, WA, USA). Blood was collected in BD Vacutainer™ SST tubes (Thermo, MA, USA), incubated at room temperature for 10 min, and centrifuged for 10 min at 4000 rpm at 4 °C. Separated serum samples were used for measuring the type II collagen IgG in serum according to the manufacturer’s instructions.

### Enzyme-linked immunosorbent assay (ELISA)

ELISA was conducted for the measurement of IL-6 and TNF-alpha levels in the serum. After euthanasia, blood was collected in BD Vacutainer™ SST tubes (Thermo, MA, USA), incubated at room temperature for 10 min, and centrifuged for 10 min at 4000 rpm at 4 °C. Separated serum samples were used for measuring the IL-6 and TNF-alpha levels. The ELISA kits used were Mouse IL-6 DuoSet ELISA (DY406–05, R&D systems, MN, USA) and Mouse TNF-alpha DuoSet ELISA (DY410–05, R&D systems, MN, USA). All experiments were conducted according to the manufacturer’s instructions.

### Hematoxylin & Eosin staining

Hind limbs were harvested from DBA/1 mice, fixed overnight in 10% NBF (Sigma Aldrich, St. Louis, MO, USA) and then paraffinized with paraplast (39,603,002, LEICA biosystems, Wetzlar, Germany), xylene (Sigma Aldrich, 534,056, St. Louis, MO, USA), and diluted ethanol. Paraffinized hind limb samples were cut into 5 μm sections using a microtome (LEICA biosystems, Wetzlar, Germany). The paraffin embedded sections were deparaffinized with xylene and hydrated with water and diluted ethanol, and then subjected to hematoxylin & eosin staining.

### Blood chemistry analysis

Blood chemistry analysis was conducted using the FUJI DRI-CHEM 4000i analyzer (Fujifilm, Tokyo, Japan), according to the manufacturer’s instructions. Blood collected in BD Vacutainer™ SST tube (Thermo, MA, USA), was incubated at room temperature for 20 min and then centrifuged for 10 min at 4000 rpm at 4 °C. The separated serum was used for blood chemistry analysis (BUN, blood urine nitrogen; Cre, creatinine; AST, aspartate serum transferase; ALT, alanine amino transferase).

### Statistical analysis

Results were expressed as the mean ± SEM. Between group comparisons were conducted using one-way ANOVA by SPSS (SPSS Inc., IL, USA), followed by Tukey’s post hoc test. A value of *p* < 0.05 was considered significant.

One-way ANOVA with SPSS was performed to compare the groups based on rheumatoid arthritis score, followed by Tukey-Kramer’s multiple comparison test. *P* values < 0.05 were considered statistically significant.

## Results

### Isolation and structural elucidation

**Fig. 1:**
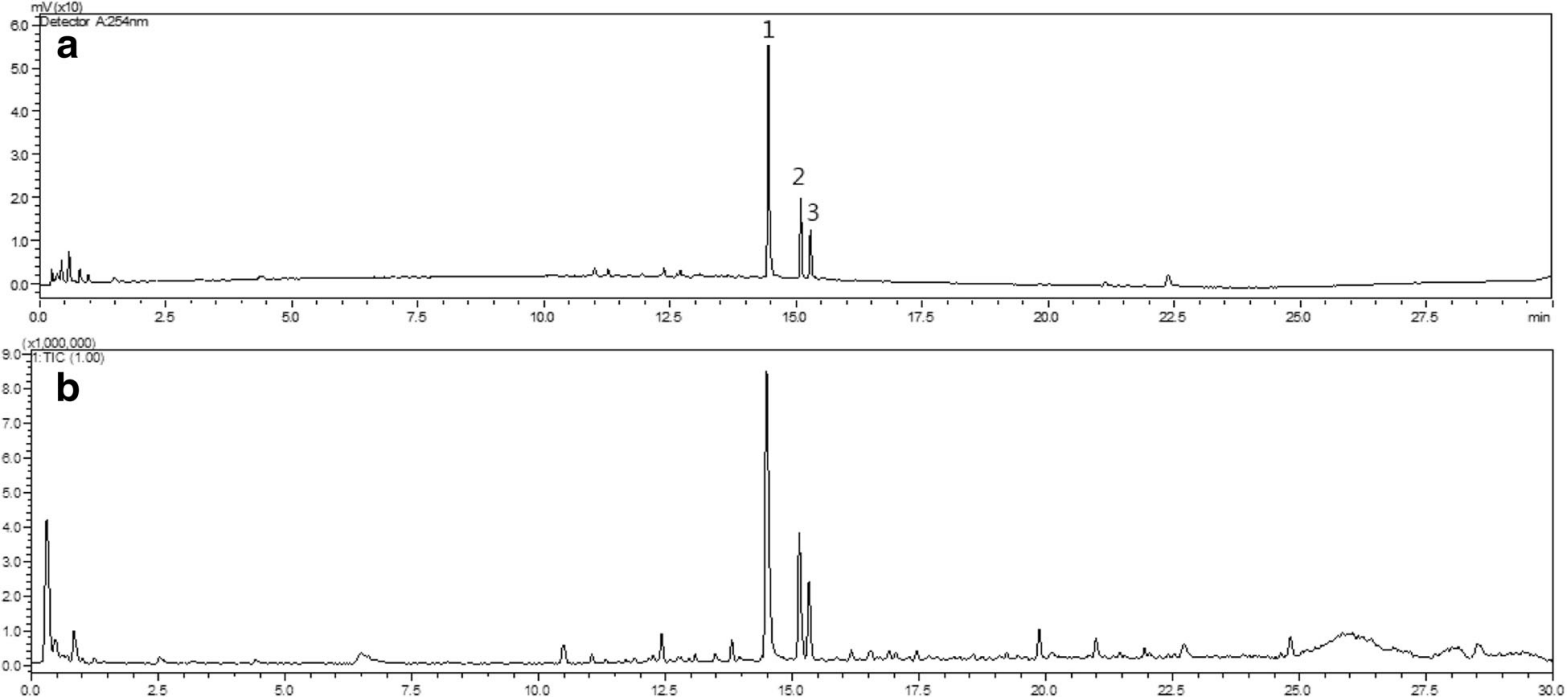
LC-IT-TOF MS chromatograms of *Saururus chinensis* leaves. (**a**) HPLC chromatogram at 254 nm; (**b**) Total ion chromatogram in the positive ion mode

The HPLC chromatogram and total ion chromatogram of *Saururus chinensis* leaves is shown in Fig. 1. Compound 1 was detected at 14.5 min, *m/z* 479.08 [M + H]^+^, MS/MS fragment ion occurred at *m/z* 303.05 [M-glucuronic acid+H]^+^. Compound 2 was detected at 15.1 min, *m/z* 611.16 [M + H]^+^, fragment ions occurred at *m/z* 449.10 [M-glu + H]^+^, *m/z* 303.05 [M-glu-rham+H]^+^. Compound 3 was found at 15.3 min, *m/z* 479.08 [M + H]^+^, MS/MS fragment ion at *m/z* 303.05 [M-rham+H]^+^. These compounds have been reported in a previous study on *Saururus chinensis* leaves but a standard preparation of compound 2 is not commercially available. To obtain the standard compound we performed isolation and identification. Compound 1 was identified as miquelianin and compound 2 was identified as quercetin 3-*O*-(2”-*O*-β-glucopyranosyl)-α-rhamnopyranoside upon comparison of spectroscopic data with literature; the purity was confirmed via LC-IT-TOF-MS. Compound **3** was identified as quercitrin based on comparison of its mass spectroscopic data with that of the purchased standard compound [26, 27].

### Qualitative analysis of *Saururus chinensis* leaves

**Fig. 2:**
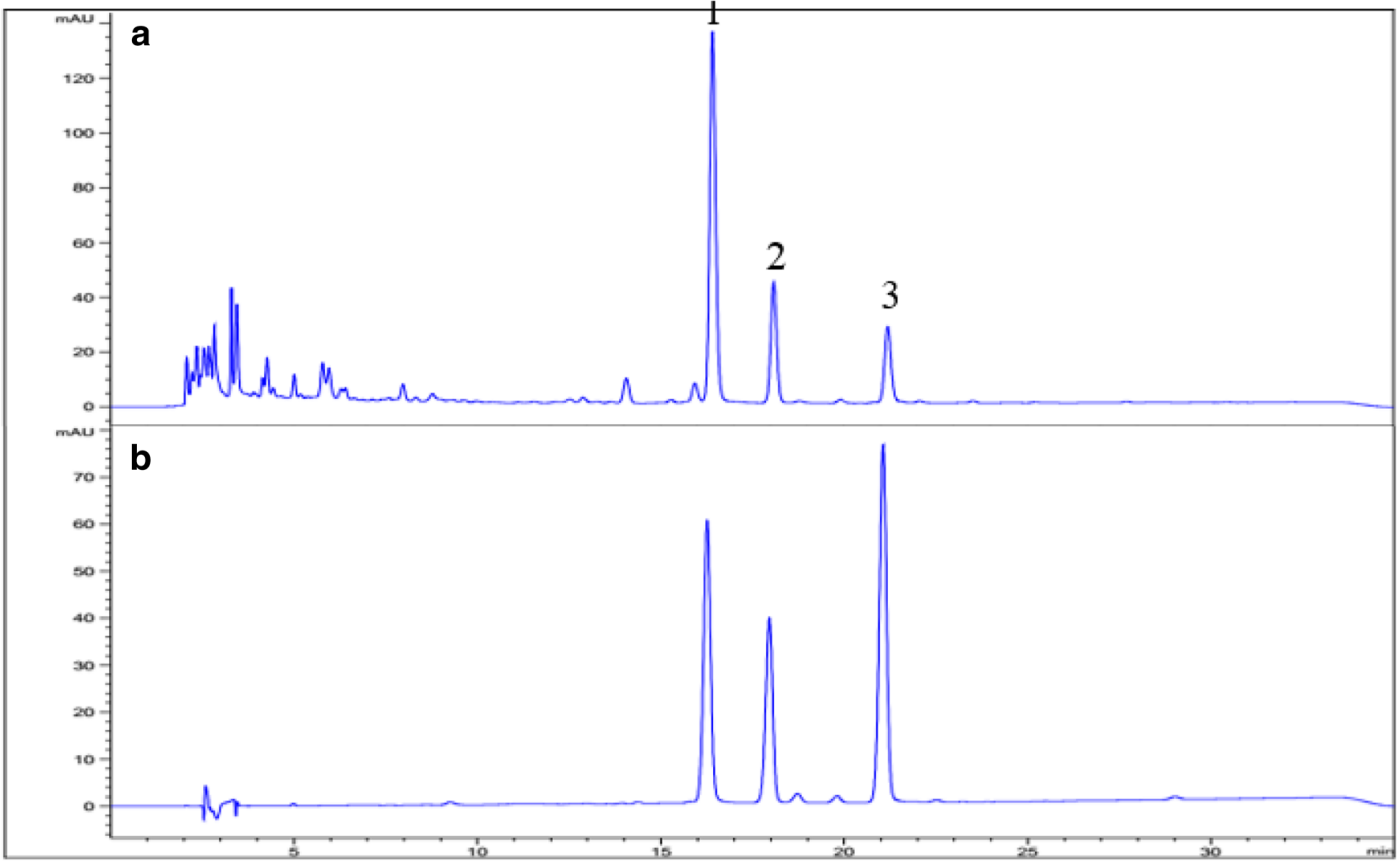
HPLC chromatograms of SHW (**a**) and standard solution (**b**). 1, Miquelianin; 2, Q-3-(2″-glu)-rham; 3, Quercitrin

HPLC chromatograms are shown in Fig. 2. Three compounds were detected at *t*_*R*_ 16.3 min, 18.0 min, and 21.1 min without interference. All of the regressions coefficients were r^2^ > 0.999 and the calibration curve showed good linearity of the detector over the range, respectively (Table 2). Several extraction solvents were examined in order to obtain satisfactory extraction efficiency. Most of the flavonoid glycosides were more soluble in a mixture of organic solvent:water rather than in a single solvent [28]. In this study, 30–50% methanol showed similar results for the three compounds, but the best proportion of the extracting solvent was chosen as 70% methanol (Table 3) with sonication for 60 min. As a result, the content of miquelianin was 56.4 ± 0.52 mg/g, that of Q-3-(2″-glu)-rham was 7.75 ± 0.08 mg/g, and the of quercitrin was 3.17 ± 0.02 mg/g.

**Table 2:** Linearities, regression equation of compound 1–3

	Regression equation	R^2^	Linear range (ug/mL)
Miquelianin	y = 8.3173x-50.936	0.9999	420–26.25
Q-3-(2″-glu)-rham	y = 18.359x-14.669	0.9999	105–6.563
Quercitrin	y = 31.668x + 4.057	1	58.75–3.672

**Table 3:** Concentration of compounds from extraction solvent

Analytes	Extraction solvent	Contents (mg/g)			Mean	SD	%RSD
		1	2	3			
Miquelianin	H_2_O	53.478	53.142	53.216	53.279	0.176	0.331
	30% MeOH	54.802	54.614	54.369	54.595	0.217	0.398
	50% MeOH	56.722	55.673	55.474	55.959	0.670	1.198
	70% MeOH	56.448	56.897	55.853	56.399	0.524	0.928
	MeOH	35.938	35.414	35.179	35.510	0.388	1.093
Q-3-(2″-glu)-rham	H_2_O	7.340	7.285	7.307	7.311	0.027	0.373
	30% MeOH	7.506	7.483	7.441	7.477	0.033	0.439
	50% MeOH	7.779	7.682	7.609	7.690	0.086	1.114
	70% MeOH	7.763	7.831	7.669	7.754	0.081	1.050
	MeOH	5.532	5.502	5.468	5.501	0.032	0.584
Quercitrin	H_2_O	2.698	2.726	2.751	2.725	0.027	0.979
	30% MeOH	2.850	2.805	2.951	2.869	0.075	2.608
	50% MeOH	3.155	3.183	3.110	3.149	0.037	1.181
	70% MeOH	3.168	3.189	3.158	3.172	0.016	0.4926
	MeOH	2.312	2.354	2.349	2.338	0.023	0.988

### SHW administration decreased the serum level of IL-6 and TNF-alpha and did not influence toxicity markers of the liver and kidneys in CIA animal models

**Fig. 3:**
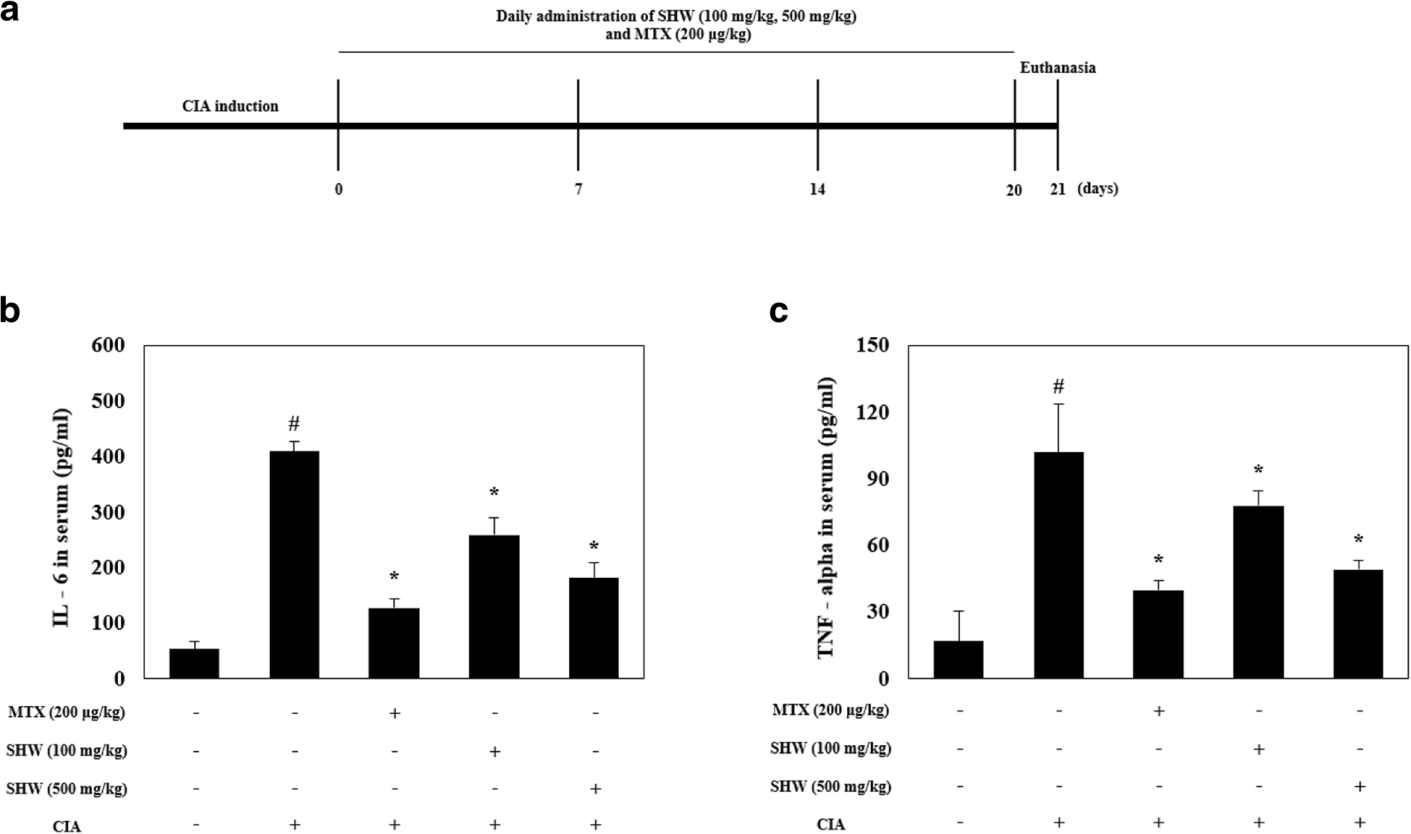
SHW administration decreased the serum level of IL-6 and TNF-alpha and did not influence the liver and kidney toxicity markers in the CIA animal models. (**a**) Experimental schedule. (**b**-**c**) Mice were separated into 5 groups (Control; *n* = 7, CIA; *n* = 7, CIA + MTX; *n* = 7, CIA + 100 mg/kg SHW; *n* = 7, CIA + 500 mg/kg SHW; *n* = 7). IL-6 and TNF-alpha levels in serum were analyzed by ELISA. Data are presented as the mean ± SEM (*n* = 7). ^#^*p* < 0.05, versus normal control group; ^*^p < 0.05, versus CIA group. Between groups comparisons were conducted using one-way ANOVA with Tukey’s post hoc test. CIA, collagen induced arthritis; MTX, methotrexate; SHW, water extract of *Saururus chinensis* leaves

To confirm the toxicity of SHW on the liver and kidneys, we evaluated the commonly used toxicity markers including BUN (blood urine nitrogen), Cre (creatinine), AST (aspartate serum transferase), and ALT (alanine amino transferase). The experimental schedule is indicated in a diagram (Fig. 3A). BUN and Cre are typically used as indicators of kidney function, whereas AST and ALT are used as biochemical indicators of liver function [29]. In this study, SHW administration slightly increases BUN in DBA/1 mice, but did not affect Cre, ALT, and AST (data not shown).

To confirm the anti-inflammatory effect of SHW administration, SHW was orally administered to CIA animal models. Administration of 500 mg/kg SHW reduced the serum level of IL-6 (Fig. 3B), but it was not more efficient than the CIA + MTX groups. As shown in Fig. 3C, the serum level of TNF-alpha was decreased by administration of 500 mg/kg SHW. However, administration of 500 mg/kg SHW was not more efficient than the administration of 200 μg/kg MTX (Fig. 3C).

### Effect of SHW administration on CIA-induced swelling and erythema in the hind limbs

**Fig. 4:**
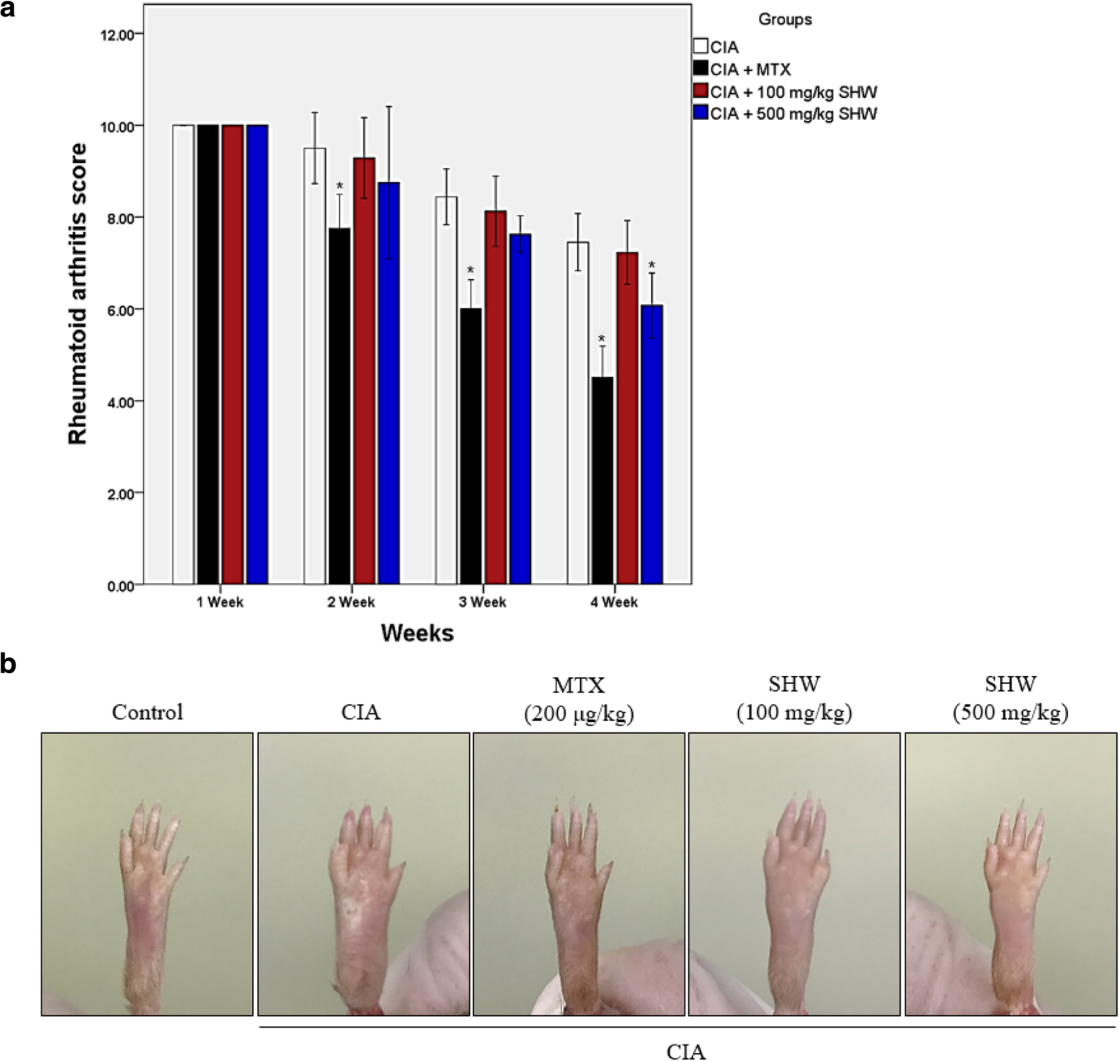
Effect of SHW administration on CIA-induced swelling and erythema of hind limbs. (**a**-**b**) Mice were separated into 5 groups (Control; *n* = 7, CIA; *n* = 7, CIA + MTX; *n* = 7, CIA + 100 mg/kg SHW; *n* = 7, CIA + 500 mg/kg SHW; *n* = 7). Morphological analysis was carried out on hind limbs. Rheumatoid arthritis score was assessed weekly beginning from 21 days of the second immunization, by examiners over three times per week. Clinical assessment was scored as follows: 0 = symptomless, 2 = erythema, 4 = mild swelling and erythema, 6 = mild swelling, erythema from the tarsals to the ankle, 8 = moderate swelling, erythema from the metatarsal joints to the ankle, 10 = severe swelling and erythema from the foot to the ankle. The X axis indicates Weeks. Data are presented as the mean ± SEM (*n* = 7). ^*^p < 0.05, versus the CIA group by one-way ANOVA with Tukey-Kramer’s multiple comparison test. CIA, collagen induced arthritis; MTX, methotrexate; SHW, water extract of *Saururus chinensis* leaves

To confirm the protective effect of SHW administration, morphological analysis of the hind limbs was performed. Treatment with SHW at 500 mg/kg significantly reduced the swelling and erythema scores compared to those in the CIA groups. However, administration of 500 mg/kg SHW in CIA animal models was not more efficient than the administration of 200 μg/kg MTX (Fig. 4A). As shown Fig. 4B, the swollen hind limbs induced by CIA were diminished by both 200 μg/kg MTX and 500 mg/kg SHW administration (Fig. 4B).

### Effect of SHW administration on type II collagen IgG levels and infiltration of inflammatory cells in the synovial membrane

**Fig. 5:**
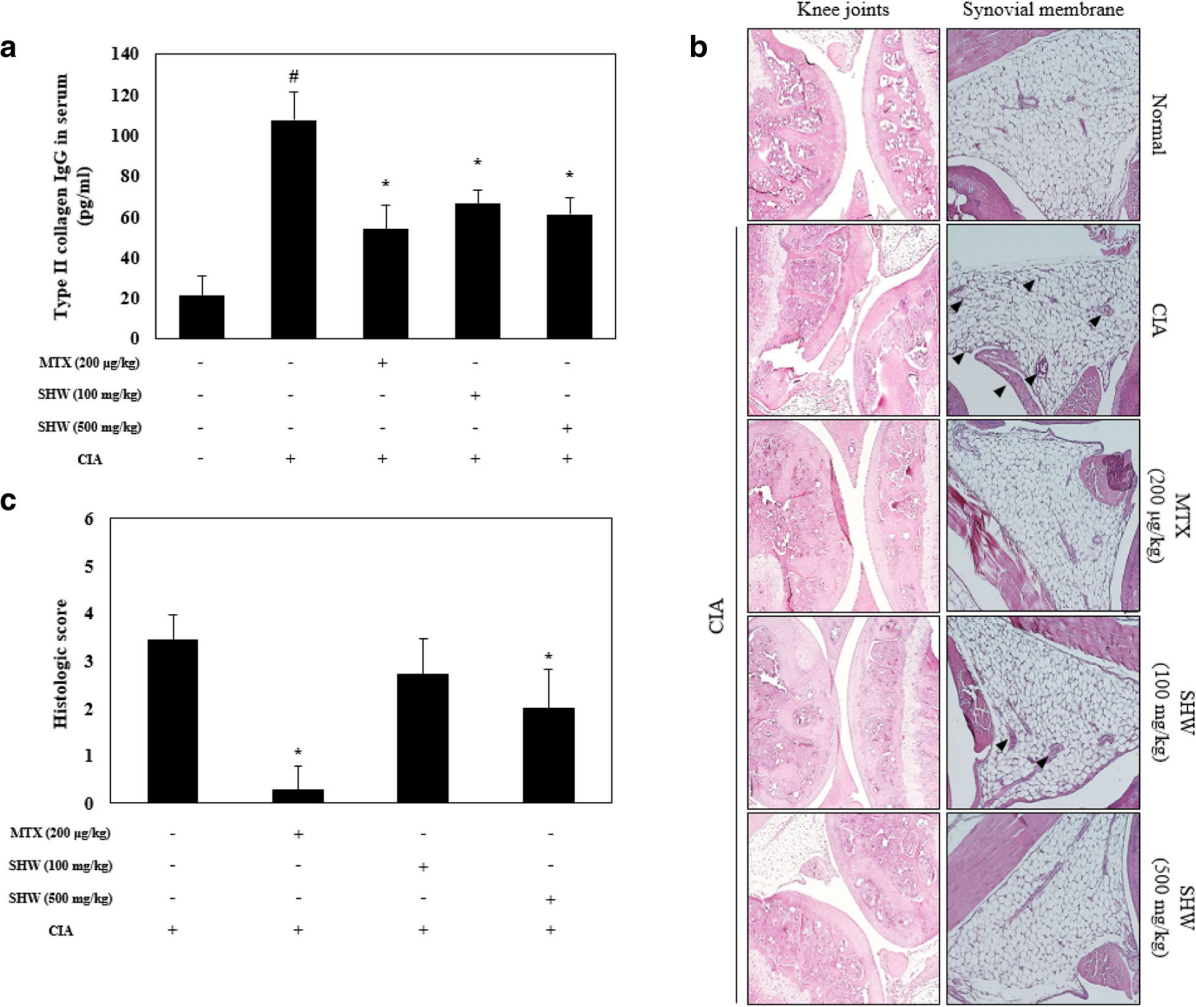
Effect of SHW administration on type II collagen IgG levels and infiltration of inflammatory cells in the synovial membrane. Mice were separated into 5 groups (Control; *n* = 7, CIA; *n* = 7, CIA + MTX; *n* = 7, CIA + 100 mg/kg SHW; *n* = 7, CIA + 500 mg/kg SHW; *n* = 7). (**a**) Type II collagen IgG in serum was analyzed using ELISA. (**b**) Representative images were stained with hematoxylin and eosin (H&E). Infiltration of inflammatory cells is indicated with black arrowheads. (**c**) Histological scores were determined on H&E stained sections in different groups of mice. Histologic scoring of inflammation and bone erosion was performed by three independent observers. Data are presented as the mean ± SEM (*n* = 7). ^#^*p* < 0.05, versus normal control group; ^*^p < 0.05, versus CIA group. Between group comparisons were conducted using one-way ANOVA with Tukey’s post hoc test. CIA, collagen induced arthritis; MTX, methotrexate; SHW, water extract of *Saururus chinensis* leaves

Finally, to elucidate the effect of SHW on CIA animal models, we confirmed the histological features and the serum levels of type II collagen IgG. As shown Fig. 5A, the serum level of type II collagen IgG was increased in the CIA groups. However, it was decreased by 200 μg/kg MTX, 100 mg/kg SHW, and 500 mg/kg SHW administration. Moreover, the inflammatory response was diminished by SHW administration in the synovial membrane and knee joints (Fig. 5B). These data suggest that SHW administration may reduce the inflammatory response in CIA animal models.

## Discussion

This study demonstrated the anti-inflammatory effect of SHW administration in CIA animal models through improved inflammatory responses such as elevated IL-6, TNF-alpha, and type II collagen IgG in serum, as well as swelling of the hind limbs. SHW administration could inhibit the development of arthritis. In addition, safety evaluation of medicinal herbs used as traditional medicine in Korea, China and Japan [30–32]. We evaluated the safety of SHW using toxicity marker such as BUN, Cre, AST, and ALT. As previously mentioned, BUN and Cre are commonly used as indicators of renal function [29]. BUN and Cre are nitrogenous end products, BUN is the metabolite derived from dietary and tissue protein. Similarly, Cre is a product of muscle creatinine metabolism. Both are distributed throughout the total body fluids, and are increased during kidney dysfunction such as nephrotoxicity and diabetic nephropathy [33].

IL-6 (Interleukin-6) is pivotal cytokine that mediates RA pathogenesis, and is found in the synovial fluid and serum of RA patients. Moreover, IL-6 promotes joint destruction by stimulating neutrophil infiltration, osteoclast maturation, and pannus formation [34]. TNF-alpha (Tumor necrosis factor alpha) is a pleiotropic cytokine in RA. It is significantly increased in RA synovial tissue, but not in osteoarthritis synovial tissue [35]. Thus, we confirmed the beneficial effect of SHW administration on the IL-6 and TNF alpha. Based on our results, the serum levels of IL-6 and TNF alpha in the CIA animal models were statistically increased upon collagen administration, but this increase was diminished by SHW administration at 500 mg/kg (Fig. 3B and C). Recently, Meng et al. reported that anti-inflammatory effects of *Saururus chinensis* Baill. in murine macrophages regulating heme oxygenase-1 [36]. Moreover, various reports revealed that *Saururus chinensis* Baill. has anti-inflammatory effects [37–39]. This evidence supports the current results and suggests that SHW administration may suppress the pathophysiological features of RA.

The pathological and immunological characteristics induced by CIA administration are similar to those observed in RA in humans [40]. Our results demonstrate that treatment with 500 mg/mg SHW decreased the swelling in hind limbs (Fig. 4A and B). RA is an autoimmune disease, wherein the immune system mistakenly attacks the own body tissue, resulting in pain and swelling [35]. Our results demonstrate that SHW may affect inflammatory responses such as arteriole dilation, neutrophil migration, and expansion of capillary beds. Recent studies have revealed that type II collagen IgG is present in the synovial fluid and serum of RA patients [41]. Our results show that type II collagen IgG levels in serum are decreased upon SHW treatment (Fig. 5A). Moreover, SHW administration decreased the infiltration of inflammatory cells in the synovial membrane (Fig. 5B).

Prior to treatment, qualitative analysis was performed. Miquelianin, Q-3-(2″-glu)-rham, and quercitrin were identified in the water extract of *Saururus chinensis* leaves. Quantitative analysis of each compound performed by HPLC indicated their individual content as follows: 56.4 ± 0.52 mg/g (miquelianin), 7.75 ± 0.08 mg/g (Q-3-(2″-glu)-rham), 3.17 ± 0.02 mg/g (quercitrin).

Quercetin derivatives are the most common group of flavonoids and are associated various health benefits. Miquelianin, the major compound of SHW, is more easily and rapidly absorbed compared to quercetin and is the major bioactive component in plasma; it inhibits peroxynitrite-induced antioxidant consumption in human plasma low-density lipoprotein [42, 43]. Moreover, miquelianin interferes with the protein-protein interaction of Aβ (1–40) and Aβ (1–42) [44], and inhibits the noradrenaline-promoted invasion of MDA-MB-231 human breast cancer cell by regulating the β_2_-adrenergic signaling pathway through matrix metalloproteinase-2 (MMP2) and matrix metalloproteinase-9 (MMP9) [45]. These results could be useful for further pharmacokinetic studies on SHW and suggest that SHW treatment can be used to manage RA. However, further experiments are required to explore how SHW administration influences inflammatory signaling pathways including the NF-κB, integrin, and TNF signaling pathways.

### Conclusions

SHW administration improved the various features of arthritis and rheumatoid arthritis including elevated serum levels of IL-6, TNF-alpha, type II collagen IgG, swelling of hind limbs, and infiltration of inflammatory cells in the synovial membrane. Thus, SHW acts as therapeutic agent against arthritis and rheumatoid arthritis. However, further experiments are required to explore how SHW influences inflammatory signaling pathways such as the NF-κB and TNF signaling pathways.

## Additional file

Additional file 1: **Figure S1.** HPLC chromatograms of several samples used on this study. A~F, the water extracts of *Saururus chinensis* leaves. 1, miquelianin; 2, Q-3-(2″-glu)-rham; 3, quercitrin. (DOCX 38 kb)

## Abbreviations

ALT: Alanine amino transferase
AST: Aspartate serum transferase
BUN: Blood urine nitrogen
CFA: Complete Freund’s adjuvant
CIA: Type II collagen-induced arthritis
Cre: Creatinine
ELISA: Enzyme-linked immunosorbent assay
IACUC: Institutional animal care and use committee
IFA: Incomplete Freund’s adjuvant
IL-6: Interleukin-6,
LPS: Lipopolysaccharide
MeOH: Metahnol
MTX: Methotrexate
NIKOM: National development institute of Korean medicine
NO: Nitric oxide
NSAIDs: Non-steroidal anti-inflammatory drugs
PGE_2_: Prostaglandin E_2_,
RA: Rheumatoid arthritis
SHW: The water extract of *Saururus chinensis* leaves
TNF-alpha: Tumor necrosis factor-alpha
