# Supplementary figures and images for: Effect of Saururus chinensis leaves extract on type II collagen-induced arthritis mouse model
Source: BMC Complement Altern Med. 2019 Jan 3;19:2. doi: 10.1186/s12906-018-2418-z (PMC6318964; doi:10.1186/s12906-018-2418-z)

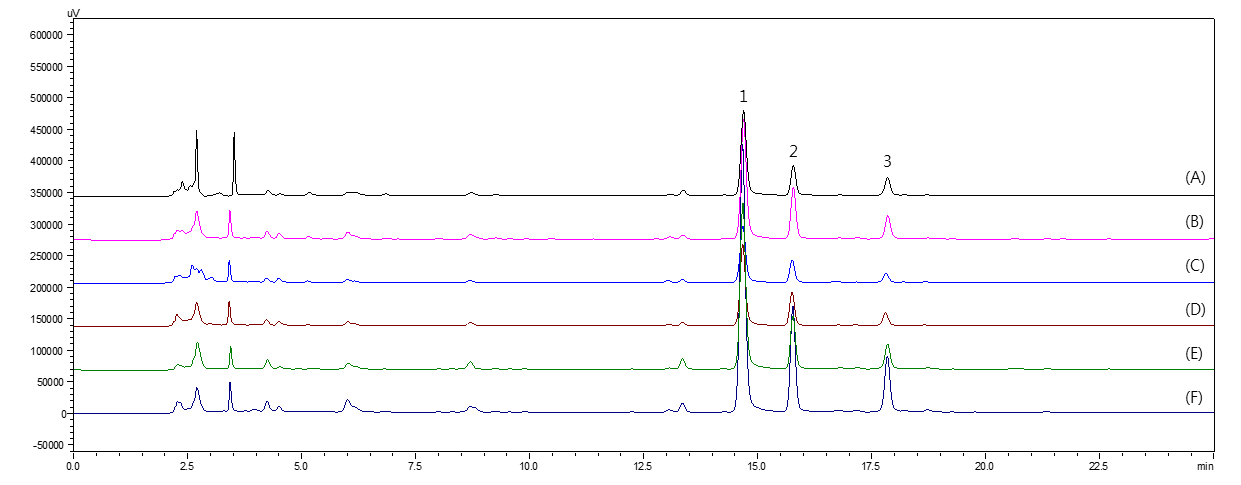

Supplement: Supplementary file 1 — Figure S1. HPLC chromatograms of several samples used on this study. A~F, the water extracts of Saururus chinensis leaves. 1, miquelianin; 2, Q-3-(2″-glu)-rham; 3, quercitrin. (DOCX 38 kb) [file 12906_2018_2418_MOESM1_ESM.docx]
